# Supplementary material for: Effect of Radiotherapy on Functional and Health-Related Quality of Life Outcomes after Jaw Reconstruction
Source: Cancers (Basel). 2022 Sep 20;14(19):4557. doi: 10.3390/cancers14194557 (PMC9559672; doi:10.3390/cancers14194557)
Supplement: Supplementary file 1 [file cancers-14-04557-s001.zip › cancers-1902238-supplementary.pdf]

Table S1 Multivariable linear regression models of the FACE-Q Head and Neck Module domains. Values represent mean differences between groups (for categorical variables) or mean differences per year increase (for the continuous variables of time since surgery and age at surgery). A negative value indicates a worse score, except for cancer worry where a positive value indicates less cancer worry.

	Appearance		Eating and drinking		Oral competence		Saliva	
**Characteristic**	**Univariable score mean difference (95% CI)**	**Multivariable regression coefficient (95% CI)**	**Univariable score difference (95% CI)**	**Multivariable regression coefficient (95% CI)**	**Univariable score difference (95% CI)**	**Multivariable regression coefficient (95% CI)**	**Univariable score difference (95% CI)**	**Multivariable regression coefficient (95% CI)**
**Time since surgery (per year increase)**	-1.8 (-4.4, 0.9)	-1.2 (-3.7, 1.3)	-0.9 (-2.8, 0.9)	-1.2 (-3.0, 0.6)	-2.8 (-5.3, -0.4)	-2.7 (-5.2, -0.3)	-2.9 (-5.1, -0.8)	-3.4 (-5.5, -1.3)
**Radiotherapy**								
No	0 [Reference]	0 [Reference]	0 [Reference]	0 [Reference]	0 [Reference]	0 [Reference]	0 [Reference]	0 [Reference]
Yes	-23.6 (-36.5, 10.6)	-24.0 (-37.0, -10.9)	-16.1 (-25.1, -7.0)	-17.4 (-26.9, -7.8)	-16.5 (-29.1, -3.8)	-15.0 (-28.0, -2.0)	-17.1 (-28.3, -6.0)	-19.9 (-31.1, -8.7)
**Bone free flap**								
No	0 [Reference]	0 [Reference]	0 [Reference]	0 [Reference]	0 [Reference]	0 [Reference]	0 [Reference]	0 [Reference]
Yes	-13.3 (-26.2, -0.5)	-6.9 (-19.8, 5.9)	-2.6 (-11.9, 6.6)	3.1 (-6.4, 12.5)	-13.0 (-25.1, -0.9)	-6.4 (-19.2, 6.4)	-0.8 (-11.9, 10.2)	8.23 (-2.8, 19.3)
**Gender**								
Male	0 [Reference]	0 [Reference]	0 [Reference]	0 [Reference]	0 [Reference]	0 [Reference]	0 [Reference]	0 [Reference]
Female	-11.5 (-24.8, 1.8)	-16.0 (-28.5, -3.6)	-3.4 (-12.9, 6.0)	-4.0 (-13.1, 5.1)	-3.7 (-16.4, 9.0)	-5.7 (-18.0, 6.6)	-5.1 (-16.3, 6.1)	-5.7 (-16.3, 4.9)
**Age at surgery (per year increase)**	0.4 (-0.1, 0.9)	0.5 (0.03, 0.9)	-0.004 (-0.3, 0.3)	-0.03 (-0.3, 0.4)	-0.002 (-0.5, 0.5)	-0.01 (-0.5, 0.4)	-0.04 (-0.5, 0.4)	-0.1 (-0.4, 0.3)

Table S1 (continued) Multivariable linear regression models of the FACE-Q Head and Neck Module domains. Values represent mean differences between groups (for categorical variables) or mean differences per year increase (for the continuous variables of time since surgery and age at surgery). A negative value indicates a worse score, except for cancer worry where a positive value indicates less cancer worry.

	Smile		Speaking		Swallowing		Appearance distress	
**Characteristic**	**Univariable score difference (95% CI)**	**Multivariable regression coefficient (95% CI)**	**Univariable score difference (95% CI)**	**Multivariable regression coefficient (95% CI)**	**Univariable score difference (95% CI)**	**Multivariable regression coefficient (95% CI)**	**Univariable score difference (95% CI)**	**Multivariable regression coefficient (95% CI)**
**Time since surgery (per year increase)**	-1.2 (-3.5, 1.1)	-1.2 (-3.5, 1.0)	-2.8 (-5.5, -0.05)	-2.7 (-5.4, 0.002)	-1.8 (-4.0, 0.3)	-2.4 (-4.5, -0.3)	-1.0 (-3.8, 1.8)	-0.5 (-3.3, 2.3)
**Radiotherapy**								
No	0 [Reference]	0 [Reference]	0 [Reference]	0 [Reference]	0 [Reference]	0 [Reference]	0 [Reference]	0 [Reference]
Yes	-16.1 (-27.7, -4.4)	-18.6 (-30.5, -6.6)	-22.9 (-36.5, -9.3)	-25.1 (-38.8, -11.5)	-17.8 (-28.5, -7.1)	-20.3 (-31.4, -9.2)	-9.8 (-24.2, 4.5)	-11.1 (-25.7, 3.5)
**Bone free flap**								
No	0 [Reference]	0 [Reference]	0 [Reference]	0 [Reference]	0 [Reference]	0 [Reference]	0 [Reference]	0 [Reference]
Yes	-1.9 (-13.4, 9.7)	8.3 (-5.3, 21.9)	-3.5 (-17.1, 10.1)	8.3 (-5.3, 21.9)	0.8 (-10.0, 11.6)	9.5 (-1.5, 20.4)	-6.5 (-20.3, 7.2)	-4.3 (-18.7, 10.2)
**Gender**								
Male	0 [Reference]	0 [Reference]	0 [Reference]	0 [Reference]	0 [Reference]	0 [Reference]	0 [Reference]	0 [Reference]
Female	-9.0 (-20.6, 2.7)	2.8 (-10.1, 15.8)	5.4 (-8.4, 19.2)	2.8 (-10.1, 15.8)	2.2 (-8.9, 13.2)	2.4 (-8.2, 12.9)	-16.1 (-29.8, -2.3)	-19.3 (-33.1, -5.4)
**Age at surgery (per year increase)**	0.3 (-0.1, 0.7)	0.5 (0.1, 1.0)	0.5 (0.04, 1.0)	0.5 (0.06, 1.0)	0.05 (-0.4, 0.5)	-0.02 (-0.4, 0.4)	0.3 (-0.2, 0.8)	0.4 (-0.1, 0.9)

Table S1 (continued) Multivariable linear regression models of the FACE-Q Head and Neck Module domains. Values represent mean differences between groups (for categorical variables) or mean differences per year increase (for the continuous variables of time since surgery and age at surgery). A negative value indicates a worse score, except for cancer worry where a positive value indicates less cancer worry.

	**Drooling distress**		**Eating and drinking distress**		**Smiling distress**		**Speaking distress**	
**Characteristic**	**Univariable score difference (95% CI)**	**Multivariable regression coefficient (95% CI)**	**Univariable score difference (95% CI)**	**Multivariable regression coefficient (95% CI)**	**Univariable score difference (95% CI)**	**Multivariable regression coefficient (95% CI)**	**Univariable score difference (95% CI)**	**Multivariable regression coefficient (95% CI)**
**Time since surgery (per year increase)**	-2.1 (-4.9, 0.6)	-2.3 (-5.1, 0.5)	-0.05 (-3.1, 3.0)	-0.7 (-3.7, 2.4)	-1.2 (-3.7, 1.4)	-1.0 (-3.5, 1.6)	-1.3 (-3.6, 0.9)	-1.0 (-3.2, 1.3)
**Radiotherapy**								
No	0 [Reference]	0 [Reference]	0 [Reference]	0 [Reference]	0 [Reference]	0 [Reference]	0 [Reference]	0 [Reference]
Yes	-12.4 (-26.8, 1.9)	-13.5 (28.2, 1.3)	-20.5 (-34.5, -6.6)	-23.0 (-37.7, 8.4)	-7.5 (-20.2, 5.2)	-10.5 (23.1, 2.2)	-13.7 (-25.1, -2.3)	-15.1 (-26.7, -3.6)
**Bone free flap**								
No	0 [Reference]	0 [Reference]	0 [Reference]	0 [Reference]	0 [Reference]	0 [Reference]	0 [Reference]	0 [Reference]
Yes	-6.8 (-20.6, 7.0)	-1.5 (-16.1, 13.1)	1.6 (-12.3, 15.5)	7.3 (-7.2, 21.8)	-0.2 (-12.3, 11.9)	1.6 (-10.9, 14.0)	-4.6 (-15.7, 6.5)	0.7 (-10.8, 12.2)
**Gender**								
Male	0 [Reference]	0 [Reference]	0 [Reference]	0 [Reference]	0 [Reference]	0 [Reference]	0 [Reference]	0 [Reference]
Female	-15.1 (-28.9, -1.2)	-17.0 (-31.0, -3.0)	-6.8 (-20.9, 7.3)	-6.0 (-20.0, 7.9)	-11.1 (-23.3, 1.0)	-12.4 (-24.4, -0.4)	-0.8 (-12.1, 10.5)	-3.0 (-14.0, 7.9)
**Age at surgery (per year increase)**	-0.2 (-0.7, 0.3)	-0.1 (-0.6, 0.4)	-0.03 (-0.5, 0.5)	-0.03 (-0.5, 0.5)	0.6 (0.1, 1.0)	0.6 (0.2, 1.1)	0.5 (0.1, 0.9)	0.6 (0.2, 1.0)

Table S1 (continued) Multivariable linear regression models of the FACE-Q Head and Neck Module domains. Values represent mean differences between groups (for categorical variables) or mean differences per year increase (for the continuous variables of time since surgery and age at surgery). A negative value indicates a worse score, except for cancer worry where a positive value indicates less cancer worry.

	**Cancer worry**		**Information**	
**Characteristic**	**Univariable score difference (95% CI)**	**Multivariable regression coefficient (95% CI)**	**Univariable score difference (95% CI)**	**Multivariable regression coefficient (95% CI)**
**Time since surgery (per year increase)**	0.08 (-2.1, 2.3)	-0.3 (-2.5, 1.9)	-1.8 (-3.5, -0.1)	-1.9 (-3.6, -0.2)
**Radiotherapy**				
No	0 [Reference]	0 [Reference]	0 [Reference]	0 [Reference]
Yes	1.4 (-9.5, 12.3)	3.4 (-7.7, 14.4)	-10.7 (-19.5, -1.9)	-11.6 (-20.7, -2.5)
**Bone free flap**				
No	0 [Reference]	0 [Reference]	0 [Reference]	0 [Reference]
Yes	-0.4 (-10.8, 10.0)	-1.5 (-12.3, 9.3)	-4.8 (-13.5, 3.9)	0.1 (-8.8, 9.1)
**Gender**				
Male	0 [Reference]	0 [Reference]	0 [Reference]	0 [Reference]
Female	-0.5 (-11.2, 10.1)	1.0 (-9.5, 11.5)	-2.0 (-11.0, 6.9)	-3.3 (-12.0, 5.4)
**Age at surgery (per year increase)**	-0.6 (-0.9, -0.2)	-0.6 (-1.0, -0.2)	0.1 (-0.2, 0.5)	0.1 (-0.2, 0.4)

Table S2 Multivariable linear regression models of the MD Anderson Dysphagia Inventory scales (MDADI). Values represent mean differences between groups (for categorical variables) or mean differences per year increase (for the continuous variables of time since surgery and age at surgery). A negative value indicates a worse score.

	MDADI Emotional Score		MDADI Functional Score		MDADI Physical Score		MDADI Composite Score	
**Characteristic**	**Univariable score difference (95% CI)**	**Multivariable regression coefficient (95% CI)**	**Univariable score difference (95% CI)**	**Multivariable regression coefficient (95% CI)**	**Univariable score difference (95% CI)**	**Multivariable regression coefficient (95% CI)**	**Univariable score difference (95% CI)**	**Multivariable regression coefficient (95% CI)**
**Time since surgery (per year increase)**	-0.6 (-2.5, 1.3)	-1.1 (-2.9, 0.8)	-0.3 (-2.3, 1.7)	-0.6 (-2.6, 1.4)	-1.4 (-3.3, 0.5)	-1.8 (-3.6, -0.06)	-0.9 (-2.7, 1.0)	-1.3 (-3.0, 0.5)
**Radiotherapy**								
No	0 [Reference]	0 [Reference]	0 [Reference]	0 [Reference]	0 [Reference]	0 [Reference]	0 [Reference]	0 [Reference]
Yes	-17.4 (-26.1, -8.8)	-19.9 (-29.1, -10.6)	-14.1 (-23.5, -4.8)	-18.9 (-26.8, -6.9)	-18.3 (-26.9, -9.8)	-20.8 (-29.8, -11.9)	-16.9 (-25.3, -8.5)	-19.5 (-28.3, 10.6)
**Bone free flap**								
No	0 [Reference]	0 [Reference]	0 [Reference]	0 [Reference]	0 [Reference]	0 [Reference]	0 [Reference]	0 [Reference]
Yes	-2.0 (-10.9, 7.0)	5.7 (-3.5, 14.8)	-1.5 (-10.9, 8.0)	5.4 (-4.4, 15.3)	-3.7 (-12.7, 5.3)	5.6 (-3.2, 14.5)	-2.6 (-11.3, 6.1)	5.6 (-3.2, 14.3)
**Gender**								
Male	0 [Reference]	0 [Reference]	0 [Reference]	0 [Reference]	0 [Reference]	0 [Reference]	0 [Reference]	0 [Reference]
Female	1.6 (-7.7, 10.9)	-0.3 (-9.2, 8.5)	3.1 (-6.6, 12.8)	0.9 (-8.7, 10.4)	3.8 (-5.4, 13.0)	1.3 (-7.2, 9.9)	3.0 (-6.0, 12.0)	0.7 (-7.7, 9.2)
**Age at surgery (per year increase)**	0.04 (-0.3, 0.4)	0.09 (-0.2, 0.4)	0.2 (-0.2, 0.5)	0.2 (-0.1, 0.5)	0.002 (-0.3, 0.3)	0.03 (-0.3, 0.3)	0.05 (-0.3, 0.4)	-0.1 (-0.2, 0.4)

Table S2 (continued) Multivariable linear regression models of the MD Anderson Dysphagia Inventory scales (MDADI). Values represent mean differences between groups (for categorical variables) or mean differences per year increase (for the continuous variables of time since surgery and age at surgery). A negative value indicates a worse score.

	MDADI Global Score	
**Characteristic**	**Univariable score difference (95% CI)**	**Multivariable regression coefficient (95% CI)**
**Time since surgery (per year increase)**	0.4 (-2.1, 3.0)	0.1 (-2.3, 2.5)
**Radiotherapy**		
No	0 [Reference]	0 [Reference]
Yes	-22.7 (-34.4, -11.0)	-25.7 (-38.1, -13.3)
**Bone free flap**		
No	0 [Reference]	0 [Reference]
Yes	-2.5 (-14.6, 9.6)	6.4 (-5.8, 18.7)
**Gender**		
Male	0 [Reference]	0 [Reference]
Female	5.7 (-6.7, 18.1)	2.7 (-9.1, 14.6)
**Age at surgery (per year increase)**	0.2 (-0.3, 0.6)	0.3 (-0.2, 0.7)

Table S3 Multivariable linear regression models of the Speech Handicap Index total score. Values represent mean differences between groups (for categorical variables) or mean differences per year increase (for the continuous variables of time since surgery and age at surgery). A positive value represents more speech-related problems.

	Speech Handicap Index	
**Characteristic**	**Univariable score difference (95% CI)**	**Multivariable regression coefficient (95% CI)**
**Time since surgery (per year increase)**	1.7 (-0.8, 4.2)	1.8 (-0.6, 4.3)
**Radiotherapy**		
No	0 [Reference]	0 [Reference]
Yes	14.9 (2.8, 27.0)	19.3 (7.1, 31.5)
**Bone free flap**		
No	0 [Reference]	0 [Reference]
Yes	0.7 (-11.3, 12.6)	-8.8 (-20.9, 3.3)
**Gender**		
Male	0 [Reference]	0 [Reference]
Female	-7.1 (-19.4, 5.2)	-3.5 (-15.3, 8.2)
**Age at surgery (per year increase)**	-0.6 (-1.0, -0.1)	-0.6 (-1.0, -0.2)
